# The Skeletal Muscle as an Active Player Against Cancer Cachexia

**DOI:** 10.3389/fphys.2019.00041

**Published:** 2019-02-18

**Authors:** Fabio Penna, Riccardo Ballarò, Marc Beltrà, Serena De Lucia, Lorena García Castillo, Paola Costelli

**Affiliations:** Department of Clinical and Biological Sciences, Interuniversity Institute of Myology, University of Turin, Turin, Italy

**Keywords:** muscle wasting, protein turnover, energy metabolism, myogenesis, adaptive response, oxidative stress

## Abstract

The management of cancer patients is frequently complicated by the occurrence of cachexia. This is a complex syndrome that markedly impacts on quality of life as well as on tolerance and response to anticancer treatments. Loss of body weight, wasting of both adipose tissue and skeletal muscle and reduced survival rates are among the main features of cachexia. Skeletal muscle wasting has been shown to depend, mainly at least, on the induction of protein degradation rates above physiological levels. Such hypercatabolic pattern is driven by overactivation of different intracellular proteolytic systems, among which those dependent on ubiquitin-proteasome and autophagy. Selective rather than bulk degradation of altered proteins and organelles was also proposed to occur. Within the picture described above, the muscle is frequently considered a sort of by-stander tissue where external stimuli, directly or indirectly, can poise protein metabolism toward a catabolic setting. By contrast, several observations suggest that the muscle reacts to the wasting drive imposed by cancer growth by activating different compensatory strategies that include anabolic capacity, the activation of autophagy and myogenesis. Even if muscle response is eventually ill-fated, its occurrence supports the idea that in the presence of appropriate treatments the development of cancer-induced wasting might not be an ineluctable event in tumor hosts.

## Introduction

Cachexia is a complex multiorgan syndrome that affects 50–80% of cancer patients and accounts for about 20% of cancer deaths (Argilés et al., 2014). Characteristic features of this syndrome are body weight loss, muscle wasting, adipose tissue depletion and metabolic abnormalities. The main symptoms include anorexia, anemia, asthenia and fatigue, that eventually result in severely impaired patient quality of life.

The pathogenesis of cancer cachexia is a complex phenomenon that includes nutritional changes, hypoanabolism, the onset of an overall hypercatabolic response that mainly affects proteins and lipids, chronic inflammation and altered energy metabolism. Last, but not least, the standard of care of neoplastic disease generally includes chemotherapy, further complicating the scenario. Indeed, cachectic patients very frequently cannot cope with anti-cancer treatment schedule and often require dosage limitation and/or therapy interruption, reducing both drug effectiveness and patient survival (Pin et al., 2018).

Several potential anti-cachexia drugs are currently being tested in clinical trials, but none of them has proved effective enough to be routinely applied in the clinical practice (Morley et al., 2014). In this regard, the small number of clinical trials and the poor knowledge about the pathogenesis of cancer cachexia likely account for the lack of effective treatments (Penna et al., 2016b).

The present review will discuss the mechanisms underlying cancer-induced wasting and will focus on the adaptive response set up by the skeletal muscle to the challenge posed by alterations at the systemic level. In this regard, most of the existent studies and reviews consider the skeletal muscle as a passive target of stimuli that eventually lead to reduced anabolism and/or increased catabolism. By contrast, muscle tissue attempts to strike back by modulating metabolism in order to set up a sort of defensive strategy against a hostile external environment. This is particularly relevant since understanding and potentiating such reactive behavior could improve the effectiveness of anti-cachexia treatments.

## Malnutrition

Cancer-associated malnutrition reflects into involuntary body weight loss that results from the combination of several factors such as reduced food intake, malabsorption, altered substrate utilization and increased substrate demand. The occurrence of malnutrition is highly unfavorable, since both recovery and tolerance to antineoplastic treatments are definitely improved in well-nourished than in malnourished subjects (e.g., with unintentional body weight loss >5%). Being malnutrition a potent predictor of bad outcome, the achievement of a good nutritional state in cancer patients should become a goal to be pursued in the clinical practice (Aversa et al., 2017). In this regard, helpful guidelines for the management of malnutrition in cancer patients have been set up by both the European Society of Parenteral and Enteral Nutrition (ESPEN; Arends et al., 2017a) and the Academy of Nutrition and Dietetics (AND; Thompson et al., 2017). These documents include the need of an early nutritional assessment, the relevance of nutritional counseling during the course of the disease, the definition of an appropriate and personalized nutritional support, the identification of the right moment for the adoption of such support.

Nutritional habits in cancer patients can be affected very early, resulting in latent malnutrition that becomes frankly evident while the disease progresses, accompanying the appearance of cachexia. Multiple mechanisms contribute to malnutrition, such as tumor localization at the gastrointestinal tract, pain, anxiety, malabsorption, and anorexia. While this latter is very often recognized in cancer patients, in the early phases of disease it happens to be underestimated and for this reason it might not be promptly addressed. To circumvent this possibility, nutritional behavior should be accurately assessed in all cancer patients at first diagnosis (Arends et al., 2017b), in order to pick up and evaluate even small changes from usual habits.

Humoral factors produced by the host as well as by the tumor have been proposed to work in concert to modulate patient nutritional behavior, affecting, directly or indirectly, the central regulation of appetite. In this regard, several pro-inflammatory cytokines have been shown to contribute to anorexia in cancer, *per se*, but also enhancing the availability of neuropeptides acting at the central nervous system level such as melanocortin, neuropeptide Y, or leptin (Ezeoke and Morley, 2015). Particularly relevant in this regard is ghrelin, an orexigenic hormone mainly produced in the stomach, whose levels are usually increased in cancer patients. Such increase could result from both an attempt to counteract anorexia and the onset of ghrelin resistance (Argilés et al., 2017). The resulting picture is the induction of signaling pathways involved in causing anorexia at the expense of those able to stimulate food intake, eventually leading to loss of appetite and/or increased satiety (Argilés et al., 2014).

Despite reduced food intake is an important component of body weight loss, nutrient availability being lower than normal, cancer-associated malnutrition is quite different from that occurring in subjects exposed to fasting or to caloric restriction. Indeed, cancer patients very often are no more able to appropriately modulate their metabolism to meet the lack of nutrients. As an example, caloric restriction in healthy subjects results in increased glucose and lipid mobilization, sparing proteins as much as possible. By contrast, reduced food intake in cancer patients is associated with random mobilization of substrates, setting protein metabolism toward a persistently negative nitrogen balance.

## Muscle Protein Turnover

**Figure 1:**
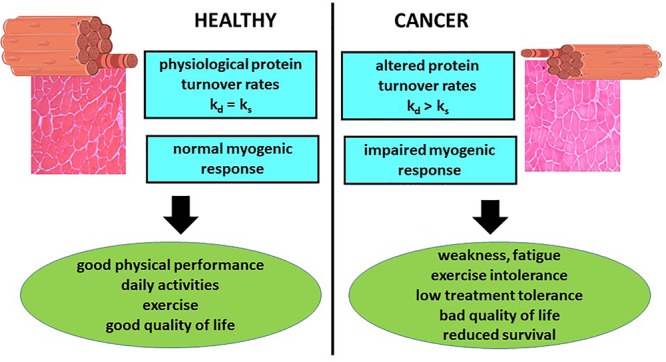
Relevance of muscle wasting to cancer patient management. The occurrence of metabolic changes that result in muscle protein hypercatabolism and impaired regeneration capacity negatively impinges on both patient quality of life and survival. k_s_ = fractional rate of protein synthesis; k_d_ = fractional rate of protein degradation.

The loss of muscle mass and function, one of the main features of cancer cachexia, markedly impairs patient quality of life and survival. In addition, muscle mass depletion has been associated with reduced tolerance to anticancer treatments (Figure 1). In addition, recent observations show that immunotherapy by checkpoint inhibitors lose effectiveness in cachectic patients, likely due to the establishment of primary resistance (Coss et al., 2018). For these reasons, an accurate estimate of muscle mass and quality should be pursued during the management of cancer patients. By contrast, still nowadays the first evaluation of a patient mainly takes into account parameters such as body weight or body mass index (BMI), that do not provide any information about body composition. Indeed, normal body weight and/or BMI could result from increased adiposity or tissue water content, *de facto* masking the occurrence of muscle mass depletion.

Protein content, the most relevant component of muscle mass, depends on the balance between rates of protein synthesis and breakdown. Physiologically speaking, disruptions of such equilibrium activate an adaptive response aimed at reaching a new homeostasis that can alternatively result in muscle hypertrophy or hypotrophy, respectively depending on the prevalence of protein synthesis or degradation (Argilés et al., 2014).

### Protein Breakdown

Intracellular protein degradation in the skeletal muscle relies on the activity of four main proteolytic pathways that depend on calpains, caspases, lysosomes, and proteasome. Results obtained in both experimental and clinical studies have clearly demonstrated that muscle wasting in cancer hosts is associated with supra-physiological activation of these proteolytic pathways (Penna et al., 2014), with particular reference to those depending on proteasome and lysosomes. These systems are involved in different aspects of intracellular protein degradation, the former breaking down short-lived and regulatory proteins, the latter being in charge of the disposal of altered organelles and structural proteins (Penna et al., 2014).

The activity of the proteasome-dependent proteolytic system depends on the availability of both ubiquitin and enzymes involved in protein substrate ubiquitylation, namely E1 (ubiquitin activating enzymes), E2 (ubiquitin conjugating enzymes) and E3 (ubiquitin ligases). As for the E3 family, some members are defined as muscle-specific. The most widely studied are MAFbx/atrogin-1 and MuRF1/TRIM63. The former is in charge of targeting proteins involved in cell cycle control, cell differentiation and cell death, while the latter mainly marks for degradation structural proteins (Argilés et al., 2014). The most recently discovered member of the muscle-specific E3 family is SMART (Milan et al., 2015). The expression levels of these muscle-specific ubiquitin ligases have been accepted as molecular markers of proteasome-dependent proteolysis and have been demonstrated to increase in different experimental models of cancer cachexia (Argilés et al., 2014). As for human studies, several reports show that in cancer patients this proteolytic system is activated above physiological levels. Of particular relevance, such enhanced activity has been observed also in non-weight losing gastric cancer patients (Bossola et al., 2003), recalling the need of early assessment of cachexia. On the other side, studies reporting unchanged levels of molecular and biochemical markers pertaining to the ubiquitin-proteasome proteolytic system in cancer patients do exist (Op den Kamp et al., 2012; Tardif et al., 2013).

The involvement of lysosomal proteolysis in muscle wasting is mainly referred to the overactivation of autophagy. This is a physiological process in charge of degrading cellular components, whose rate is increased by lack of nutrients or by the presence of damaged organelles, such as mitochondria or peroxisomes. Some years ago the discovery of autophagy-related (ATG) genes has refreshed the field, providing useful tools to investigate the process. Indeed, at least some of the proteins encoded by these genes, such as beclin 1 and LC3B are now accepted markers of autophagy. The physiological protein homeostasis in the muscle is maintained by basal autophagy, in view of its role in the routine clearance of wasting products such as altered proteins and organelles. Disruption of autophagy has been shown to be associated with progressive muscle derangements, such as those occurring in mice lacking the Atg7 or the OPA1 genes (Masiero et al., 2009; Tezze et al., 2017) or carrying the BCL2 AAA mutation (He et al., 2012). On the other side, markers of autophagy are overexpressed in several muscle wasting-associated states such as denervation and fasting, suggesting that stress-induced autophagy is activated above physiological levels in these diseases. Consistently, the induction of autophagy in the skeletal muscle of both tumor-bearing animals and cancer patients is demonstrated by several reports (Penna et al., 2013; Tardif et al., 2013; Aversa et al., 2016; Pigna et al., 2016). However, despite autophagic flux is increased, the process does not reach its final step with complete cargo degradation, as indicated by the observation that autophagosomes accumulate in the muscle of cancer hosts, likely due to lysosomal engulfment (Penna et al., 2013; Aversa et al., 2016; Pigna et al., 2016). On the whole, these observations demonstrate that the relevance of autophagy, enhanced or inhibited, to muscle wasting can be significantly different, according to the specific situation. In other words, both excessive and defective autophagy are unwanted scenarios, and treatments impinging on these modality of protein degradation should be aimed at maintaining/restoring a physiologic autophagic flux.

Myofibrillar protein degradation depends on the preliminary disruption of myofilaments. Such preventive cleavage cannot be performed either by proteasome or lysosomes and has been proposed to involve other proteases, such as caspases and calpains (Penna et al., 2014). Consistently, these latter are upregulated and overactivated in the skeletal muscle of tumor-bearing animals (Llovera et al., 1995; Costelli et al., 2002; Pin et al., 2017) and of cancer patients (Smith et al., 2011).

Unfolded protein response (UPR) is important for the maintenance of skeletal muscle mass in adults. Because PERK regulates protein folding and calcium homeostasis, it is important to investigate how these functions of PERK are affected in skeletal muscle of smPERK-KO mice. How the activation of PERK is regulated in conditions of muscle growth and atrophy and whether modulation of its activity can improve skeletal muscle mass in various catabolic states is also an important area of future research.

### Protein Synthesis

While the enhancement of muscle protein breakdown rates in cancer cachexia is clearly demonstrated, the same does not apply to protein synthesis, reduced, normal, or increased rates being reported (Argilés et al., 2014). As for experimental cachexia, the generally short time frame from tumor implantation and animal death frequently results in impaired anabolic capacity, at least in the skeletal muscle, although contrasting observations have been reported. As an example, results obtained in mice bearing the C26 or the LLC tumors show a reduction of muscle protein synthesis rates, that cannot be restored by treatments able to improve muscle mass (Toledo et al., 2016a; Nissinen et al., 2018). On the other side, protein synthesis rates close to those of controls have been observed in rats implanted with the Yoshida AH-130 hepatoma (Costelli et al., 1993). Further variability can be observed when tissues different from muscle are taken into consideration. As an example, enhanced pro-synthetic capacity has been measured in the liver of tumor-bearing animals, likely due to the increased demand of acute phase proteins. These observations put in evidence the limitations of the experimental models, where cachexia frequently develops markedly faster than in cancer patients.

The modulation of protein synthesis rates in cancer patients is less clear. Some old studies, but also a recent one, have reported that muscle protein synthesis is reduced in human pathology (Emery et al., 1984; Hanson et al., 2017). By contrast, another research group shows that pancreatic cancer patients are able to improve baseline protein synthesis rates, that result poised at values higher than those measured in healthy subjects (van Dijk et al., 2015). Between these opposites, several studies report muscle protein synthesis rates comparable to those of controls or of non-weight losing cancer patients (Engelen et al., 2016).

Stimulation of muscle protein synthesis has been proposed as a mean to improve muscle phenotype in cancer cachexia. Most of the attempts, mainly unsuccessful, have involved nutritional interventions or molecular tools aimed at improving the muscle anabolic capacity. Indeed, advanced cancer patients treated with both parenteral and enteral nutrition, including or not the supplementation with amino acids, do not show any improvement in muscle protein synthesis (Engelen et al., 2016). Similar results have been obtained in studies attempting to activate muscle protein anabolism by means of both drugs or genetic tools (Costelli et al., 2006; Penna et al., 2010a). Recent observations, however, show that there is the possibility to exploit a sort of anabolic window in cancer patients (see below).

## Energy Metabolism

The vast majority of cancer patients are hypermetabolic, presenting with resting energy expenditure (REE) higher than normal (Vazeille et al., 2017). The underlying tumor-driven mechanisms are still unclear, however released mediators and/or pro-inflammatory stimuli rather than tumor burden, that is usually quite low with respect to patient body weight (Purcell et al., 2016), are likely involved. A recent study describes the occurrence of hypermetabolism associated with body weight loss, inflammation, altered energy balance and low performance status in about 50% out of 390 cancer patients at first diagnosis, e.g., in the absence of any anticancer treatment (Vazeille et al., 2017). As stated above, metabolic alterations are an early feature in cancer patients. Indeed, the study by Vazeille et al. (2017) shows that about half of the hypermetabolic patients are non weight-losing and present with good performance status. Consistently, normal metabolism is rapidly restored in bladder cancer patients after surgery, while body weight recovery is markedly delayed (Tobert et al., 2017). These observations, coupled to those reported above on skeletal muscle protein depletion, stress the need of tools allowing an early detection of metabolic modulations in cancer hosts.

Additional mechanisms leading to increased REE in cancer patients may depend on alterations in thermogenesis. The tissues mainly in charge of this process are the brown adipose tissue (BAT) and the skeletal muscle. Both of them express high amounts of uncoupling proteins (UCPs), whose levels have been shown to further increase in tumor-bearing animals and cancer patients (Collins et al., 2002; Bing, 2011). In the last few years, white adipocytes have been proposed to convert into brown adipocyte-like (beige) cells. This process, known as ‘browning,’ relies on increased UCP1 expression, resulting in a shift of mitochondrial activity from ATP to heat production, increasing both lipolysis and energy expenditure (Argilés et al., 2014).

In addition to increased expenditure, also reduced energy production, due to undernutrition, decreased fat free mass and low physical activity, participates to generate the negative energy balance that frequently occurs in cancer patients. In this regard, in the last few years particular emphasis has been given to mitochondrial alterations occurring in the skeletal muscle, these organelles being the main source of energy production.

Muscle mitochondria in tumor-bearing animals show ultrastructural alterations (Shum et al., 2012; Fontes-Oliveira et al., 2013; Pin et al., 2015; Shum et al., 2018) associated with uncoupling, leading to reduced oxidative capacity (Julienne et al., 2012; Tzika et al., 2013). Such impairment can be associated with the systemic inflammatory response occurring in cachexia, as suggested by observations showing that activation of the transcription factor NF-κB decreases muscle oxidative capacity and down-regulates mitochondrial biogenesis (Julienne et al., 2012). In addition, altered mitochondrial dynamics (fusion and fission) and biogenesis have been reported in the ApcMin/+ mice, characterized by high circulating levels of IL-6 (White et al., 2012). Moreover, mitochondria alterations, in addition to impinge on energy production, also favor the onset of oxidative stress, both working as catabolism-inducing stimuli.

## Oxidative Stress

The intracellular sources of oxidative species such as reactive oxygen and nitrogen species (ROS/RNS) are organelles such as mitochondria and sarcoplasmic reticulum, and enzymes, in particular the nicotinamide adenine dinucleotide phosphate oxidase and the xanthine oxidase. The occurrence of oxidative stress depends on an altered balance between the production of ROS/RNS and the activity of the intracellular antioxidant systems. An altered redox homeostasis may exert important effects on the integrity of biological macromolecules, with potential consequences on both cell survival and metabolism (Gomez-Cabrera et al., 2009). In this regard, muscle wasting in cancer hosts has been associated with increased ROS/RNS levels and with enhanced oxidative damage to lipids and proteins (Mastrocola et al., 2008; Sullivan-Gunn et al., 2011; Salazar-Degracia et al., 2018). At the muscle level, these feature have also been correlated with increased protein breakdown rates as well as with the activation of the inflammatory response (Gomes-Marcondes and Tisdale, 2002; Mastrocola et al., 2008; Puig-Vilanova et al., 2015).

The increase of oxidative species in cachectic subjects may rely on several mechanisms among which pro-inflammatory cytokines, antineoplastic treatments and the loss of coupling between phosphorylation and oxidation in mitochondria. As reported above, several intracellular signaling pathways are regulated, partially at least, by ROS/RNS. Particularly relevant are those leading to activation of the redox-sensitive transcription factors NF-κB and AP-1. The former, in particular, has been shown to contribute to impaired myogenesis (see below) and to enhance the expression of molecules pertaining to the intracellular proteolytic machinery. In this regard, oxidative stress has been proposed to impinge on the activation of Ca^2+^-dependent proteolysis in the presence of Ca^2+^ overload, on caspase-3 dependent induction of proteasome activity and on up-regulation of muscle-specific ubiquitin ligases (Ábrigo et al., 2018). Moreover, a lot of evidence suggests that also autophagic degradation can be regulated by oxidative species, mainly acting on signaling pathways dependent on p38 or PI3K/Akt (Ábrigo et al., 2018).

Finally, redox homeostasis in the cell largely depends on mitochondria ‘well-being.’ Indeed, altered or damaged mitochondria fail to produce energy and may become both an uncontrolled source of ROS and a target for such oxidant species. The presence of altered mitochondria is an additional pro-catabolic stimulus, resulting in activation of mitophagy. While the goal of such activation is to get rid of useless organelles, the result is a reduction of muscle mitochondria abundance, that frequently in cachectic subjects is not associated with enhanced mitochondrial biogenesis, also in view of the reduced energy availability (Ábrigo et al., 2018).

## Cytokines and Hormones

More than 150 years ago, Rudolf Virchow proposed that carcinogenesis requires a pro-inflammatory milieu, mainly on the basis that morphological analysis repeatedly reports infiltration of the tumor stroma by inflammatory cells (Liu et al., 2017). About one century later, the discovery of cytokines and of the role that these mediators play in tumor progression has provided an additional support to the relevance of inflammation to carcinogenesis. In addition, the same mediators have been shown to significantly contribute to the pathogenesis of cancer cachexia (Argilés et al., 2014).

**Figure 2:**
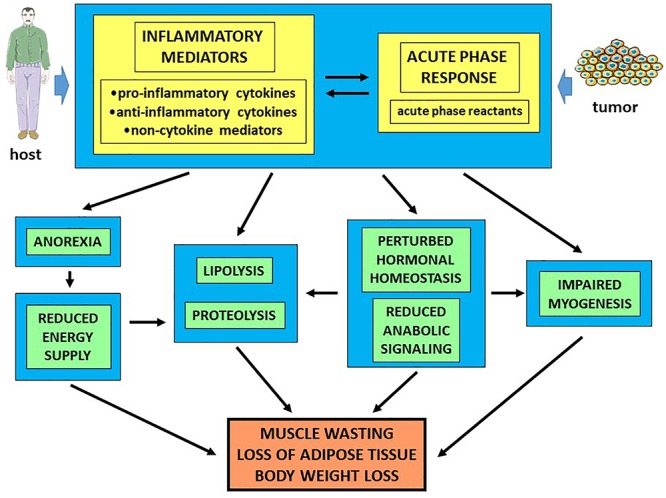
Humoral mediators of cancer cachexia. Humoral mediators differentially involved in the inflammatory response play a crucial, but probably not exclusive, role in the pathogenesis of cancer cachexia.

In the last two decades, cancer cachexia has been mainly explained as the result of systemic inflammation (acute phase response) due to the host response to tumor growth (Figure 2). Several observations support such hypothesis. Indeed, in cancer patients plasma levels of acute phase reactants, C reactive protein in particular, are positively correlated with increased REE and predict reduced survival rates. Along this line, circulating C reactive protein levels contribute to build up the Glasgow prognostic score (Fearon et al., 2006). To obtain the amino acids necessary to synthesize the acute phase proteins, the liver markedly impinges on muscle metabolism, leading to increased protein degradation and, depending on the tumor, to reduced protein synthesis rates. Finally, also the skeletal muscle of tumor-bearing mice has been shown to express mRNAs coding for several acute phase reactants (Bonetto et al., 2011), although the real meaning of this expression pattern is still unknown.

The hypercatabolic setting that characterizes cancer cachexia results, partially at least, from the complex interplay among humoral mediators, including proinflammatory cytokines, hormones and growth factors. Reciprocal regulations occur in cancer hosts, cytokines being able to impinge on the hormonal homeostasis and vice versa. High plasma levels of IL-6, TNFα, and γ-INF have been observed in both tumor-bearing animals and cachectic cancer patients (Tisdale, 2010). Several cytokines, among which TNFα, have been shown to modulate peripheral insulin sensitivity, with a mechanism that affects the activation of the insulin receptor and of down-stream signaling molecules. In addition, TNFα also down-regulates the signaling pathways dependent on the insulin-like growth factor (IGF)-1, reducing muscle anabolic capacity and enhancing the pro-catabolic stimuli (Tisdale, 2010). Few years ago, TGFβ deriving from bone resorption due to metastatic disease has been shown to significantly contribute to cancer-induced muscle wasting (Waning et al., 2015). Quite recently, TGF-β and TNFα have been shown to mediate the expression of the zinc transporter ZIP14, that is overexpressed in the skeletal muscle of both cachectic tumor-bearing animals and patients affected by metastatic cancer and that plays a causative role in muscle wasting (Wang et al., 2018).

The relevance of cytokines to cachexia has been demonstrated by studies showing that their administration to healthy animals results in skeletal muscle atrophy (Tisdale, 2010) as well as in alterations of lipid metabolism associated with down-regulation of lipoprotein lipase activity, induction of the hormone-sensitive lipase and stimulation of hepatic lipogenesis (Tisdale, 2009). Most of these effects also occur in tumor-bearing animals, and can be prevented treating the animals with specific anti-cytokine antibodies (Tisdale, 2009). Despite these observations, however, there is still no clear-cut evidence that anti-cytokine strategies can be useful to manage cachexia in cancer patients.

In addition to acute phase reactants, hormones and cytokines, other mediators have been proposed to contribute to the onset and progression of cancer cachexia. As an example, fat-derived leptin has been shown to inhibit both food intake and adipose tissue mass in healthy animals, although its circulating levels do not increase in both cancer patients and tumor-bearing animals, suggesting that its role in cachexia is not a causative one (Argilés et al., 2014). Among the mediators possibly involved in causing adipose tissue depletion in cachexia, the lipid mobilizing factor, increased in the circulation of cachectic cancer patients (Bing, 2011), has been shown to induce triglyceride disassembly by stimulating the activity of both the adipose triglyceride lipase and the hormone sensitive lipase, and to upregulate UCP2 expression in liver and muscle (Bing, 2011). Several reports have shown that the expression of myostatin, a molecule belonging to the TGFβ family and endowed with inhibitory activity on muscle enlargement, is enhanced in the muscle of tumor-bearing animals, although its relevance to muscle wasting in cancer hosts has not been completely defined. In this regard, recent observations show that myostatin can be secreted by the BAT, leading to impaired mTOR signaling in fast myofibers. As a result, the expression levels of several proteins, among which components of the mitochondrial electron transport chain, are reduced, affecting the energy balance (Kong et al., 2018). Several observations report the causal involvement of other TGFβ family members such as activins, and on the basis of these findings, drugs able to interfere with the activin receptor, which is also shared by myostatin, are currently under investigation as potential tools to manage cancer cachexia (Hatakeyama et al., 2016; Nissinen et al., 2018).

Recent reports suggest that receptors of the Toll-like family (TLRs) can be involved in mediating muscle wasting in cancer cachexia. In particular, TLR4 appears to directly activate muscle protein breakdown in mice hosting the Lewis lung carcinoma. Consistently, TLR4 inhibition exerts a protective effect against muscle wasting, likely by reducing the production of pro-inflammatory cytokines such as TNFα and IL-6 (Zhang et al., 2017a). In addition, the activation of TLR4 has also been shown to depend on Hsp70 and Hsp90 that reach the muscle through exosomes released by the tumor (Zhang et al., 2017b). Similarly, the activation of TLR7 by miR-21 contained into tumor-derived extracellular vesicles has been proposed to trigger apoptosis in cultured myoblasts, potentially contributing to the defective myogenesis reported in cancer cachexia (He et al., 2014).

## Myogenesis

Myogenesis is a physiological process induced during embryonal development and susceptible of activation in the adult after skeletal muscle injury, where it is more properly defined as regeneration. Irrespective of the trigger, muscle fiber degeneration following damage implies the recruitment and activation of satellite cells, stem cells resident in the muscle underneath myofiber basal lamina, that proliferate and fuse with existing fibers to restore the original muscle mass. When regeneration is triggered by chronic pathologies such as muscle dystrophies, satellite cell pool can be exhausted, leading to substitution of muscle with connective tissue.

The amount of satellite cells is not the same in every muscle type. As an example, it is higher in oxidative than in glycolytic muscles, respectively characterized by slow and fast contraction (Yin et al., 2013). In addition, satellite cell population is heterogeneous in terms of both myogenic potential and ability to perform the asymmetric division, the hallmark of stemness. In addition to satellite cells, several other cell types have been recognized as endowed with myogenic potential, such as mesoangioblasts and PW1^+^ cells. Finally, an important role during myogenesis/regeneration is played by fibroadipogenic progenitors (FAPs), cells that are unable to differentiate to muscle, but that are crucial to sustain myogenic precursors during proliferation and differentiation (Costamagna et al., 2015).

The activation of myogenesis in the adult strictly relies on modulations of the muscle microenvironment, that are different depending on the regenerative stimulus, e.g., acute damage or chronic diseases. Particularly relevant, in this regard, are the interactions occurring among immune cells, humoral mediators and myogenic precursors. Indeed, the recruitment of pro-inflammatory (M1) macrophages in the injured muscle is concomitant with satellite cell activation. However, the subsequent step of myogenic differentiation (satellite cell fusion with existing myofibers) strictly requires the induction of M1 cell apoptosis and their replacement with anti-inflammatory (M2) macrophages (St Pierre and Tidball, 1994). This transition must take place with an accurate timing (M1: 1–2 days post-injury; M2: 4 days post-injury) in order to ensure damage repair and the down-regulation of injury-induced inflammatory response. A crucial role in such process is played by cytokines, produced by macrophages but also deriving from other cellular sources. The production of TNFα and IL-10 allows the shift from pro-inflammatory to anti-inflammatory milieu, killing FAPs and M1 macrophages and allowing the recruitment of M2 cells (Perdiguero et al., 2011; Fiore et al., 2016). In addition, IL-10 also promotes the differentiation of myogenic precursors different from satellite cells (Bosurgi et al., 2012). If cytokine production does not conform to the correct regeneration schedule, however, the process can be markedly impaired. As an example, if TNFα is produced earlier than days 3–4 post-injury, regeneration is impaired due to persistently cycling satellite cells (Guttridge et al., 2000; Bakkar et al., 2008). The other way round, if TNFα is not produced at the right moment, FAPs will not die and, taking advantage of anti-inflammatory cytokines such as IL-10 and TGFβ, will result in the overproduction of extracellular matrix, eventually leading to muscle fibrosis (Fiore et al., 2016).

Alterations in the regenerative process have been proposed to contribute to muscle wasting occurring in several chronic diseases such as dystrophies, myopathies, autoimmune diseases, and cancer. Focusing on this latter, experimental studies performed in mice hosting the C26 tumor have shown in the muscle increased expression of Pax7, a marker of satellite cell activation, and reduced levels of myogenin, an indicator of ongoing differentiation. Similar observations have been reported in cancer patients (Ramamoorthy et al., 2009; Penna et al., 2010b; He et al., 2013). Consistently, *in vivo* regeneration is delayed in the muscle of tumor-bearing mice, with a mechanism that has been proposed to involve a persistent activation of the transcription factor NF-κB (He et al., 2013), and the increased phosphorylation of the stress kinase ERK (Penna et al., 2010b).

Recent observations have shown that overexpression of Twist1, a transcription factor associated with the malignant progression of several tumors, in myogenic precursors leads to skeletal muscle hypotrophy. Such a pattern is associated with increased myostatin expression in Twist1^+^ satellite cells. The authors hypothesize that in satellite cells Twist1 drives both myostatin synthesis and secretion. The released myostatin targets myofibers that respond by increasing Twist1 expression, resulting in muscle wasting (Parajuli et al., 2018). Of interest, Twist1 levels higher than in healthy mice have been observed in the skeletal muscle of animals bearing different experimental tumors. Not only, if Twist1 expression is abrogated in satellite cells of tumor hosts, cancer-induced muscle wasting appears prevented (Parajuli et al., 2018). Finally, the zinc transporter ZIP14, recently shown to contribute to muscle wasting in cancer hosts, has also been proposed to interfere with regeneration, since it is induced in satellite cells isolated from cachectic muscles. Consistently, differentiation is impaired in ZIP14 overexpressing C2C12 myoblasts in the presence of zinc (Wang et al., 2018).

## The Skeletal Muscle Strikes Back: Adaptive Mechanisms to Face the Wasting Drive

Looking at the history of skeletal muscle biology, it is quite evident that while the relevance of this tissue to the organism life has been understood very early, for quite a long time the muscle has been considered as a sort of protein-containing black box, that receives nutrients, oxygen and signals from outside and returns the contractile activity necessary for breathing, moving, heart beating, etc. Only in the last few decades the idea that the skeletal muscle is indeed a metabolically active tissue able to release mediators and to influence, directly or indirectly, other body compartments has been gaining a growing consensus. However, still nowadays, when pathological muscle wasting is addressed, the general approach is to take into consideration, mainly at least, only alterations that take place in the extra-muscle body compartments and that target the muscle. This occurs, for example, when cancer-induced systemic inflammation and/or malnutrition are evoked to explain muscle wasting in cachexia. For sure, the role played by these features is overall recognized; what is lacking to this picture, however, is the response set up by the muscle to the systemic alterations. The general view, in this regard, is that the muscle passively suffers this sort of ‘aggression’ coming from the extra-muscle environment, being unable to face the external catabolic stimuli that eventually lead to the loss of muscle mass and function. However, several evidence suggest that the skeletal muscle is indeed able to set up compensatory, although eventually unsuccessful, strategies attempting to counteract cancer-induced hypercatabolism and hypoanabolism. Along this line, understanding these strategies will be highly valuable in order to design protocols aimed to support the muscle in the struggle against the wasting drive.

### Muscle Anabolic Response

The above reported concept that slimming in cachexia is different from that due to caloric restriction is based on the observation that the latter is adopted in a physiological metabolic environment, while the former occurs in the presence of marked abnormalities, such as inflammation and hypermetabolism. In particular, cancer hosts might not properly activate protein synthesis following an increased nutrient loading, a condition that has been defined as ‘anabolic resistance.’ Its occurrence seriously questions the usefulness of anabolism-promoting strategies: if the patient cannot activate protein synthesis, no nutritional or pro-anabolic intervention will ever be successful. In the last few years, however, the idea that cancer hosts still maintain the ability to activate anabolism, at least until cachexia does not reach the refractory stage, has gained a growing consensus (Prado et al., 2013; Engelen et al., 2016; Antoun and Raynard, 2018).

Several observations derived from both experimental and clinical studies support this hypothesis. Muscle wasting in tumor-bearing animals is unexpectedly associated with unchanged or increased levels of molecules involved in the protein synthetic machinery. As an example, the PI3K-Akt-mTOR signaling is poised toward activation in mice implanted with the C26 tumor (Penna et al., 2010a), while protein synthesis rates are unchanged with respect to controls in rats hosting the Yoshida AH-130 hepatoma or the MCA sarcoma (Tessitore et al., 1987; Stallion et al., 1995). As for human pathology, habitual myofibrillar protein synthesis rates in gastric cancer patients, either weight stable or weight losing, have been reported to be comparable to control subjects (MacDonald et al., 2015). Consistently, in the muscle of cancer patients, the pAkt/Akt ratio and the levels of pGSK3β, both molecular markers of an intracellular pro-anabolic setting, are comparable to control levels or even increased (Aversa et al., 2012; Stephens et al., 2015). A study performed on patients affected by non-small cell lung cancer has shown the activation of a normal anabolic response after an euglycemic, hyperinsulinemic clamp associated with amino acid supplementation (Winter et al., 2012). Similarly, a significantly high anabolic response independent from body weight loss, muscle mass depletion and the occurrence of inflammation, has been obtained feeding lung cancer patients with an essential amino acid integration (Engelen et al., 2015). Finally, the association of conventional nutritional support with high leucine, fish oil and carbohydrate supplementation has enhanced the muscle anabolic response in advanced cancer patients (Deutz et al., 2011). On the whole, these observations support the idea that, if adequately supported, cancer patients might benefit from timely adopted nutritional, better if protein enriched, interventions.

In addition to the supply of amino acids, mainly mere ‘bricks’ to build up new proteins, nutritional supplements can also be useful in order to modulate both muscle and extra-muscle environments. This applies, for example, when molecules able to reduce inflammation are included in the nutritional formula. Polyunsaturated fatty acids (PUFAs) belonging to the ω-3 series have revealed promising in this regard: a recent meta-analysis based on the screening of a huge number of clinical trials involving chemotherapy-treated cancer patients has shown that oral nutritional supplementation exerts beneficial effects only when ω-3 PUFAs are included in the formulation (de van der Schueren et al., 2018). It is still debated if ω-3 PUFA supplementation also results in improved patient outcome. While several studies support such possibility (reviewed in Laviano et al., 2018), contrasting evidence do exist. In this regard, no improvement of overall survival has been reported in a study investigating the effects of ω-3 PUFA supplementation in cachectic gastrointestinal cancer patients treated with chemotherapy (Shirai et al., 2017).

Strategies aimed at counteracting members of the TGFβ family such as myostatin and activin have been tested. Particularly interesting appear the studies involving soluble activin receptor type IIB and antibodies directed against the activin II receptor (Bimargumab). In tumor-bearing mice the former has been reported to increase survival and muscle wasting as well as to improve the anabolic and anti-catabolic effect of formoterol (Toledo et al., 2016b; Nissinen et al., 2018). Similarly, Bimagrumab has proved effective in preventing muscle atrophy induced by glucocorticoids or cancer (Lach-Trifilieff et al., 2014; Hatakeyama et al., 2016). Clinical studies have been performed or are currently ongoing in order to test humanized antibodies targeting the activin IIB receptor. At present, the results available demonstrate that lean mass and muscle strength are increased in antibody-receiving volunteers (Becker et al., 2015).

Another exploitable strategy to improve anabolism is the administration of ghrelin. In this regard, increased circulating ghrelin markedly modulates protein and energy metabolism (Ezquerro et al., 2017). Consistently, tumor-bearing animals treated with ghrelin show increased food intake, improved body composition and increased tolerance to anti-cancer drugs (Graf and Garcia, 2017). Since ghrelin administration might also exert undesirable effects, ghrelin analogs have been produced and are currently studied in clinical trials. Among the most promising, anamorelin has been shown to improve body composition and muscle function in non-small cell lung cancer patients (Takayama et al., 2016). By contrast, other studies have clearly shown that anamorelin fails to improve motor function in cancer patients (Temel et al., 2016), likely suggesting that muscle function cannot be rescued by adopting single agent pharmacological strategies. Subsequent investigations have shown that, in selected groups of patients, anamorelin also leads to improved performance status (Katakami et al., 2018). Other appetite stimulants such as macimorelin and a synthetic human ghrelin are actually under investigation (Argilés et al., 2017).

### Enhanced Intracellular Protein Breakdown as a Salvage Pathway

An adequate protein homeostasis, e.g., a balance between protein synthesis and degradation rates, is required to properly maintain cell functions and to prevent the onset and progression of diseases. In this regard, the observation that protein catabolism is generally increased in several conditions associated with muscle mass depletion, including cancer cachexia, has characterized this arm of protein metabolism with a negative connotation. However, normal cell function cannot disregard the presence of an adequate rate of protein turnover, meaning that the production of new proteins must be paralleled by the disassembly of pre-existing damaged or misfolded proteins. This is true in the whole organism and in the muscle in particular, where several myopathies arise due to the absence of physiological levels of protein catabolism (Bell et al., 2016). Indeed, the physiological rate of protein degradation in the skeletal muscle is considerably high, provided that this tissue is constantly exposed to damaging events such as mechanical stretch, force generation, and oxidative stress.

The observation that muscle intracellular proteolytic systems are activated above physiological levels in cancer cachexia (see above) has provided the basis for different experimental approaches aimed at contrasting the onset and progression of muscle mass depletion. As an example, genetic inhibition of muscle-specific ubiquitin ligases has been shown to effectively counteract the loss of muscle proteins (Rom and Reznick, 2016). Such an approach, however, has not yet been validated for the clinical use. Another tool has come from the discovery of rather specific proteasome inhibitors such as bortezomib. While this drug is widely used to treat hematologic malignancies, studies performed on experimental models of cancer cachexia have demonstrated that specific proteasome inhibition does not improve muscle phenotype (Penna et al., 2016a). The lack of effectiveness of the pharmacological approaches aimed at counteracting proteasome-dependent proteolysis likely depend on the compensatory activity set up by the other intracellular proteolytic systems, further supporting the idea that if muscle protein breakdown is overactivated in cancer cachexia, this does not happen by chance. In other words, such activation above physiological levels is likely part of an adaptive response due to the steadily increased abundance of damaged/unwanted proteins, ultimately aimed at maintaining vital functions. In this regard, targeting the mechanisms leading to protein alteration rather than protein degradation systems should be pursued.

Particularly relevant in terms of protein homeostasis maintenance is the proteolytic system that relies on autophagy. Enhancement of the autophagic-lysosomal protein degradation has been demonstrated in several conditions characterized by muscle wasting, including myopathies and cancer cachexia (Penna et al., 2014), suggesting that limiting the activation of this proteolytic system could be the goal to achieve in order to prevent or at least delay the loss of muscle mass and function. Such interpretation, however, does not take into account the physiological relevance of autophagy. In this regard, observations performed on experimental animals genetically manipulated in order to obtain a muscle-specific autophagy-defective phenotype have clearly shown that autophagy is required to maintain a correct muscle morphology, a healthy mitochondrial compartment and a proper force-generating capacity (Masiero et al., 2009). Consistently with these observations, gene strategies aimed at silencing Beclin-1, a key player of autophagosome formation, have proved ineffective in preventing the reduction of myofiber atrophy in mice hosting the C26 tumor (Penna et al., unpublished). In addition, frankly cachectic C26-bearing mice treated with pharmacological inhibitors of the autophagic flux do not survive (Penna et al., 2013). This observation suggests that autophagy in the muscle of the C26 hosts is activated above physiological levels also in order to provide substrates that are no more available from the usual sources. In this regard, at a certain point at least, such overactivation becomes an adaptive response that is crucial to maintain the body homeostasis or to support a new homeostatic level in stressful conditions. Partially consistent with these observations are the results reported by a study showing that stimulation of stress-induced autophagy obtained through mTOR inhibition improves muscle phenotype in tumor hosts (Pigna et al., 2016). However, long term treatment with mTOR inhibitors in patients has been shown to result in muscle wasting (Gyawali et al., 2016). Finally, overactivation of autophagy obtained through TP53INP2/Dor overexpression was shown to exacerbate the loss of muscle mass observed in experimental diabetes, confirming that both direct inhibition and stimulation of autophagy are detrimental in wasting conditions (Sala et al., 2014).

On the whole, these observations support the idea that modulations of autophagy can be good treatment options to manage muscle wasting in cancer cachexia only if they succeed in maintaining the physiological flux. In other words, too less autophagy is as detrimental as too much autophagy. Finally, the observation reported above clearly define that the prevention or delay of the onset and progression of muscle wasting in cancer cachexia cannot be obtained by targeting one specific proteolytic system. By contrast, acting on the mechanisms that activate the hypercatabolic drive appears a more promising strategy. Just as an example, β_2_-adrenergic agonists have been demonstrated to effectively improve muscle phenotype in tumor-bearing animals as well as in cancer patients (Busquets et al., 2004); such protection is associated with down-regulation of both proteasome activity and autophagy (Penna et al., unpublished).

The regulation of protein hypercatabolism in muscle wasting has also been associated with the activation of the UPR due to endoplasmic reticulum (ER) stress (Afroze and Kumar, 2017; Ma et al., 2017). Indeed, increased expression of ER stress markers has been reported in denervated muscles (Yu et al., 2011) as well as in the muscle of tumor-bearing animals (Bohnert et al., 2016). Such a response has been proposed to reflect a compensatory mechanism, since inhibition of both ER stress and UPR results in the induction of muscle wasting, the more so when the muscle is already depleted (Afroze and Kumar, 2017). More recently, evidence suggesting the relevance of the PERK arm of the unfolded protein response to the maintenance of muscle mass and function has been reported (Gallot et al., 2018).

### Oxidative Stress Management

Oxidative species such as ROS and RNS have been involved in the pathogenesis of cancer-induced muscle wasting (see above). Along this line, antioxidant compounds such as vitamins C and E, α-lipoic acid, *N*-acetylcysteine, and polyphenols have been proposed as useful therapeutic tools. Polyphenols, in particular, have been shown to protect against the onset of cachexia in different types of tumors (Oelkrug et al., 2014; Gil da Costa et al., 2017), likely due to their inhibitory effect on NF-κB (Gil da Costa et al., 2017). On the other side, several pre-clinical and clinical studies have demonstrated that the anti-cachectic effects exerted by anti-oxidant drugs are poor, if not frankly detrimental (Busquets et al., 2007; Assi et al., 2016). In this regard, depending on the body compartment, tumor included, a pro-oxidant environment can be either detrimental or beneficial and anti-oxidant treatments can be helpful and exert unwanted effects at the same time.

Both systemic mediators and intramyofiber events, such as proinflammatory cytokines and mitochondrial alterations, respectively, can generate a pro-oxidant environment in the skeletal muscle of cancer hosts. However, even when muscle mass and function are significantly reduced with respect to control values, the tissue is able to activate an effective antioxidant response. Indeed, increased mRNA expression of both Cu/Zn SOD and catalase, associated with unchanged levels of protein carbonylation and malondialdehyde, can be observed in the muscle of C26-bearing mice (Assi et al., 2016; Ballarò et al., unpublished). Such antioxidant response is further enhanced when tumor-bearing animals are exposed to a moderate exercise protocol, that also results in improved muscle mass and function (Ballarò et al., unpublished).

These results suggest that, despite profound metabolic alterations occur in the muscle of cancer hosts, this tissue is still able to activate and maintain an efficient anti-oxidant response, suggesting that tissue-specific interventions able to improve such adaptation would be beneficial to prevent or delay the progression of cachexia toward the refractory phase.

### Activation of the Myogenic Response

Physiologically, the skeletal muscle activates the regeneration program to face myofiber injury. Previous observations have reported that this process is activated in the skeletal muscle of cancer hosts (see above), however, what is actually triggering regeneration in this situation is still debated. In this regard, few studies suggest that alterations of the dystrophin glycoprotein complex leading to sarcolemma leakage play a crucial role (Acharyya et al., 2005; He et al., 2013). However, the observation that an inflammatory infiltrate is lacking in the muscle of tumor-bearing animals (Berardi et al., 2008) does not support this hypothesis. Another possibility is that the activation of the regenerative response is another face of the residual anabolic capacity reported in the muscle of cancer hosts. According to this possibility, but with unknown mechanisms, the muscle could react to the hypercatabolic state by recruiting and activating myogenic precursors, attempting to counteract the loss of muscle proteins. Such a strategy is however ineffective, since for unclear reasons committed Pax7^+^ myogenic precursors do not reach the complete differentiation and accumulate in the muscle without proceeding to fuse with existing myofibers. Consistently with this hypothesis, treatments able to release the impairment of myogenesis, such as MEK inhibitors or Pax7 silencing, also result in improved muscle mass and function (Penna et al., 2010b; Prado et al., 2012; He et al., 2013; Talbert et al., 2017).

Very little is known about the mechanisms that impair complete myogenic differentiation in the muscle of cancer hosts. The first possibility is that something has changed in muscle stem cells, leading to altered differentiation capacity. However, myogenic precursors isolated from the muscle of C26-bearing mice have been shown to perfectly differentiate *in vitro* (He et al., 2013; Inaba et al., 2018; Costamagna et al., unpublished data), demonstrating that the impaired regeneration does not depend on a cell autonomous defect. Taking into consideration these results, a possible alternative is that the presence of the tumor results in the generation of a muscle microenvironment that is not permissive for complete regeneration. Along this line, the persistently increased activation of both ERK and NF-κB (see above) are likely part of the mechanism that modulates muscle microenvironment. An additional mechanism can rely on the reduced recruitment to the muscle of neutrophils, macrophages, and mesenchymal progenitors occurring in tumor-bearing animals (Inaba et al., 2018).

Basal autophagy is also required to ensure both the maintenance of satellite cell homeostasis and a proper muscle regenerative response. Indeed, autophagy is steadily activated in quiescent satellite cells in order to get rid of potentially dangerous wasting products, preserving cell ‘well-being.’ Defective autophagy results in satellite cell senescence, mainly due to the accumulation of altered mitochondria that leads to oxidative stress, eventually reducing both the stem cell pool and function. Senescent muscle stem cells are unable to be recruited and activated in response to damaging stimuli (Sousa-Victor et al., 2018). Such pattern can be reversed by restoring basal flux of autophagic degradation or by pharmacological inhibition of ROS release above physiological levels (García-Prat et al., 2016). In addition, in the regenerating muscle autophagy has been proposed to impinge on myoblast differentiation (Fiacco et al., 2016; Fortini et al., 2016). Last but not least, autophagy is responsible for providing activated satellite cells with nutrients required to sustain the increased demand to exit quiescence and entering the cell cycle (Tang and Rando, 2014). Consistently with this hypothesis, the reduced expression of TP53INP2/DOR in the skeletal muscle of tumor-bearing mice and cancer hosts (Penna et al., unpublished) suggest that indeed basal autophagy is reduced in these conditions, potentially impinging also on Pax7^+^ cells.

The activation of satellite cells is characterized also by modulations in the oxidative pattern. Quite recent studies show that genetic manipulation of Pitx2 and Pitx3 transcription factors, known to regulate the redox homeostasis during embryonal myogenesis also affects the differentiation of adult satellite cells. In particular, muscle regeneration fails in animals depleted of both factors, that are characterized by markedly increased ROS levels (L’honoré et al., 2018). The same study reports that, while ROS increase is required by satellite cell to exit from quiescence and to proceed toward differentiation, excess ROS is detrimental (L’honoré et al., 2018). Along this line, reduced oxidative potential has been reported in satellite cells isolated from muscle biopsies of cancer patients, that are also endowed with reduced ability to differentiate (Brzeszczyńska et al., 2016). There is the possibility that the same alteration of redox state applies to myogenic precursors isolated from tumor-bearing animals, partially explaining the impaired regenerative response (Penna et al., 2010b; He et al., 2013). On the other side, the oxidative stress reported in the muscle of cancer hosts could also reflect the attempt to generate an environment permissive for satellite cell activation and differentiation.

## Conclusion

Cancer cachexia arises from a complex milieu in which several factors such as metabolic alterations, malnutrition and systemic inflammation play a crucial role. Since this multiorgan syndrome is an important challenge in patient management, the definition of markers suitable to allow its early identification is actively pursued for therapeutic purposes.

**Figure 3:**
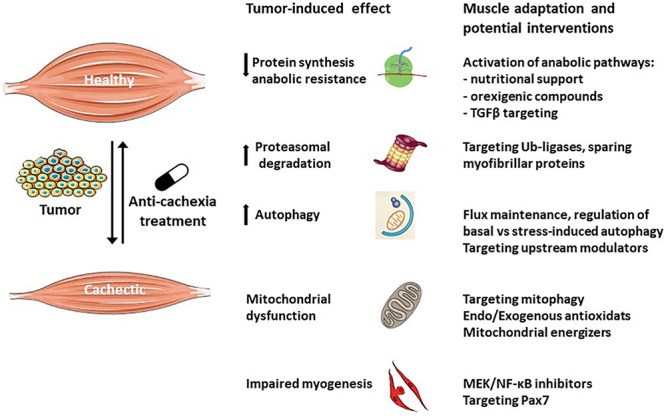
Muscle compensatory mechanisms activated in response to tumor growth. The skeletal muscle is a plastic tissue able to autonomously respond to the wasting stimuli in order to maintain the homeostasis. Muscle loss occurs as a consequence of the failure to adapt to the alterations induced by the tumor. Anti-cachexia drugs targeting specific cellular and molecular processes will boost the muscle adaptation potential, counteracting the wasting process.

Several reviews analyzed the therapeutic strategies actually available to counteract cancer cachexia (Advani et al., 2018; Di Girolamo et al., 2018; Solheim et al., 2018); while quite different approaches are adopted, all the studies agree in stating that they are far from being effective, likely because the mechanisms underlying this syndrome are only partially elucidated. Particularly relevant in this regard, is the observation that target tissues such as the skeletal muscle are able to react to the wasting stimuli activating compensatory responses (Figure 3). These counteractive strategies, however, are not potent enough and do not succeed in preventing the progression of cachexia. Along this line, the multimodal therapeutic protocol to treat cachexia could take advantage also of new approaches that, relying on understanding tissue-specific compensatory responses, contribute to support the organism effort in defeating the wasting drive.

## Author Contributions

FP prepared the first draft of the review. RB, MB, SD, and LGC contributed sections of the review. PC supervised the final version. All authors read and approved the submitted version.

## Conflict of Interest Statement

The authors declare that the research was conducted in the absence of any commercial or financial relationships that could be construed as a potential conflict of interest.
